# Discrete Kinetic Models from Funneled Energy Landscape Simulations

**DOI:** 10.1371/journal.pone.0050635

**Published:** 2012-12-12

**Authors:** Nicholas P. Schafer, Ryan M. B. Hoffman, Anat Burger, Patricio O. Craig, Elizabeth A. Komives, Peter G. Wolynes

**Affiliations:** 1 Chemistry/Biochemistry, University of California San Diego, La Jolla, California, United States of America; 2 Center for Theoretical Biological Physics, University of California San Diego, La Jolla, California, United States of America; 3 Rice University, Houston, Texas, United States of America; 4 Physics, University of California San Diego, La Jolla, California, United States of America; 5 Chemistry, Rice University, Houston, Texas, United States of America; 6 Physics and Astronomy, Rice University, Houston, Texas, United States of America; University of South Florida College of Medicine, United States of America

## Abstract

A general method for facilitating the interpretation of computer simulations of protein folding with minimally frustrated energy landscapes is detailed and applied to a designed ankyrin repeat protein (4ANK). In the method, groups of residues are assigned to foldons and these foldons are used to map the conformational space of the protein onto a set of discrete macrobasins. The free energies of the individual macrobasins are then calculated, informing practical kinetic analysis. Two simple assumptions about the universality of the rate for downhill transitions between macrobasins and the natural local connectivity between macrobasins lead to a scheme for predicting overall folding and unfolding rates, generating chevron plots under varying thermodynamic conditions, and inferring dominant kinetic folding pathways. To illustrate the approach, free energies of macrobasins were calculated from biased simulations of a non-additive structure-based model using two structurally motivated foldon definitions at the full and half ankyrin repeat resolutions. The calculated chevrons have features consistent with those measured in stopped flow chemical denaturation experiments. The dominant inferred folding pathway has an “inside-out”, nucleation-propagation like character.

## Introduction

Energy landscape theory and the principle of minimal frustration, which provide both simple models and interpretative frameworks [1], [2], have contributed greatly to our understanding of the protein folding process. Proteins have evolved to minimize the effects of roughness of their energy landscapes by ensuring a significant stability gap between the unfolded ensemble and the native state. This leads to landscapes that resemble the high-dimensional analog of a rugged funnel. Protein folding can therefore be understood as a diffusive process across a rugged, biased, and structurally correlated energy landscape with weak transient trapping. Translating the ruggedness and stability gap ideas into mathematical terms has allowed self-consistent optimization methods to learn predictive potentials from structural data [3], [4]. Coarse-grained models based directly on known protein structures have been derived that are computationally tractable, yet able to provide insight into, and generally show qualitative and often even quantitative agreement with, experimental results [5]. All-atom simulations of fast folding proteins are just now becoming reliable [6] and give results largely consistent with the rugged funnel landscape picture [7]. However, model building is only part of the challenge facing theorists working on protein folding since, even on a minimally frustrated landscape, many seemingly distinct detailed mechanisms of folding are possible.

In order to interpret raw simulation results in ways that deepen our understanding of folding, researchers can either take advantage of the connection between structure and energy implied by theory and experiment to exist for natural proteins (using the principle of minimal frustration) or try to remain agnostic as to whether such a connection exists. The former choice leads to free energy based methods that use global, structure based reaction coordinates to calculate free energy profiles [8]. This global description facilitates comparison across a wide range of systems and development of physical intuition about details of specific systems. Furthermore, these free energy based methods can be combined with semi-analytical perturbation methods [9] to extrapolate existing simulation data to new simulation conditions. The more agnostic schemes sometimes start by using approximate reaction coordinates suggested by landscape theory but often rely on clustering strategies to define macrobasins. Such agnostic schemes generally have only provided predictions of rates for each given set of simulation conditions independently, in contrast with experiments that usually scan a range of thermodynamic conditions. Such schemes thus entail a significant computational load when comparing with experiment. Recently some suggestions have emerged of how such general methods can be extended to combine data from parallel tempering simulations to yield kinetic models at arbitrary temperatures [10].

In this paper, we describe a free energy based method that can be used to derive kinetic equations that are similar to those derived using clustering based approaches but that take into account what has been learned about natural protein folding. This method maintains the attractive features of both free energy based methods using smooth reaction coordinates and clustering algorithms to provide predictions about rates and insight into folding mechanisms under a continuous range of conditions. The resulting folding mechanisms are expressed in terms of the cumulative flux through the network of macrobasins [11].

## Methods

### 1.1 Foldons and reaction coordinates

The most basic criterion for defining a hierarchy of states in a kinetic model is that a separation of time scales should exist. Dynamics within a defined macrobasin should ideally be fast compared to the interconversion between the macrobasins. Many clustering strategies attempt to directly apply this criterion to simulation data. However, for folding models based on minimally frustrated landscapes we can take advantage of the connection between structure and energy to help choose natural ways of coarse-graining a protein's conformational space without already knowing the results of the simulation. These methods are necessarily approximate, but may, in many cases, be sufficient as well as efficient. Even on a rough energy landscape, if there are correlations, geometrical distances between structures are a good guide as to the barriers between them [12].

For this study, we will define foldons as contiguous regions of primary structure that may fold independently. This corresponds to a *putative* foldon as defined by Panchenko and others, which requires the contiguous primary structural regions to be kinetically competent [13]. The word “foldon” is sometimes employed to describe the notion of cooperatively folding substructures with no constraint on primary structural contiguity [14]. Such a scheme can also be useful but the first guess that contiguous regions reconfigure most rapidly is often correct.

The study of ankyrin repeat proteins has already revealed that the choice of folding units can be non-trivial. We use the designed ankyrin repeat protein 4ANK [15] as illustration. We adopt structurally motivated schemes for defining foldons in this system, namely that each repeat, or each half repeat, is one foldon [16], [17]. For other types of proteins, different schemes may be more appropriate, and general schemes for approximate foldon assignment exist [13].

To measure the foldedness of the individual foldons, we use the reaction coordinate 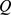 given in Equation 1.
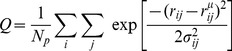
(1)In Equation 1, 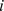 and 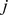 are residue indices, 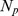 is the total number of pairs 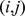, 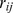 is the distance between the 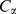 atoms of residues 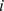 and 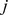, 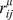 is the same distance in the experimentally determined native structure, and 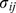 is a sequence separation dependent width. We define the degree of foldedness of a foldon as the instantaneous value 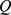 as given in Equation 1 where the summation over 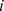 is taken over all residues within a foldon and 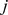 goes over all residues within the same foldon and those in native contact with residue 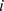 as defined by an 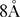 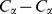 distance cutoff. 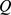 has a range between 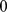 and 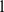, with 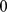 being completely unfolded and 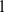 being completely folded.

### 1.2 Macrobasins and free energy calculations

For the purposes of defining a set of discrete macrobasins, we set a foldedness threshold above which a foldon may be considered essentially folded. Below this threshold, the foldon is considered to be unfolded. For the results shown in Section 3, this threshold has been set to 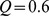. Using this scheme, any arbitrary structure from a simulation of a protein with, for example, 4 foldons can be assigned to a macrobasin such as 0101, indicating that the second and fourth (but not the first and third) foldons exceed the foldedness threshold. A protein with 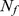 foldons therefore has 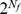 macrobasins, though not all such macrobasins would necessarily be observed in each set of simulations. This scheme is very analagous to the Ising model schemes used extensively by Munoz and Eaton [18].

We performed molecular dynamics simulations in the canonical ensemble, employing a biasing potential to umbrella sample along a global reaction coordinate 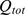, defined as the value of 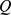 (Equation 1) obtained by summing over all unique 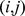 pairs. We then used the multistate Bennet acceptance ratio (MBAR) [19] to compute the relative free energies of all sampled macrobasins over a range of temperatures. MBAR is a method that can be used to combine data from multiple equilibrium simulations at different thermodynamic states to obtain unbiased free energy differences and expectation values.

### 1.3 Transition rates and kinetic equations

Before considering the transition rates between macrobasins, it is necessary to define the connectivity of the discrete macrobasin space. It is reasonable to assume that locality of dynamics would imply that each macrobasin is directly connected to other macrobasins for which only a single 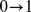 or 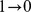 reconfiguration event is required to change the starting state into the final state. That is to say the direct transition 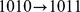 is allowed, but 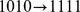 and 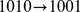 are not directly allowed because they both require two local reconfiguration events and would in all likelihood be made up of composites of the simpler local moves. This is an example of a locally connected landscape; the effects of local connectivity on folding have been discussed previously [20].

The transition rate for going from macrobasin 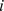 to macrobasin 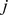, 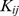, is given in Equation 2 where 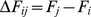 is the free energy difference between the macrobasins' free energies, 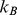 is the Boltzmann constant, 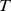 is the absolute temperature and 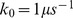 is the assumed universal downhill transition rate. A similar rate scheme was adopted by Zheng *et al.* [21] when studying Trp-Cage using stochastic simulations on a kinetic network. The value of 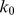 is motivated by a consideration of the ultimate speed limit of folding and measurements of the kinetics of downhill folding domains, as has emerged from numerous studies starting with the Eaton group [22]–[24]. The diagonal values of the matrix are defined so as to conserve probability, 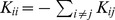, where 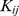 refers to the element in the 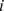
th column and the 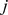
th row of matrix 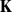.
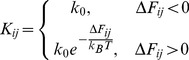
(2)From these microscopic rates it is well known how to derive the overall kinetics by diagonalizing the rate matrix [25], [26]. The set of 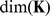 eigenvalues, 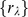, and corresponding eigenvectors, 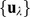, are used in Equations 3–4. The instantaneous population of state 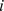 at time 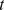 is denoted 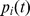. The time dependence of 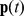, given in Equations 3 and 4, is then a function of the rate matrix, 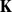, and the initial concentrations 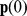 via the coefficients 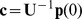 where 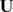 is the matrix of eigenvectors.
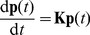
(3)

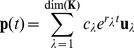
(4)


For systems obeying detailed balance the eigenvalues 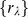 are all real and less than or equal to zero. Ordering them from largest to smallest, the resulting eigenvalue spectrum falls into two limiting scenarios [27]. If the largest non-zero eigenvalue (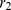) is well-separated from the next-largest eigenvalue, the system will initially rapidly relax in a multi-exponential fashion, then will be dominated by a single exponential. If several non-zero eigenvalues are all similar in magnitude, multi-exponential decays may be apparent.

The expression that we used to evaluate the cumulative flux between any two macrobasins 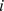 and 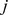 over a time interval 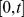 is given in Equation 5.
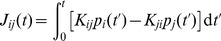
(5)We evaluated Equation 5 from an initial concentration vector corresponding to a completely folded or unfolded state 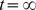, yielding equilibrium fluxes. We used the GraphViz software [28] to visualize the fluxes between each pair of directly connected macrobasins. Several examples of resulting flux diagrams are given in Section 3.

## Model

### 2.1 Hamiltonian

The model used for the simulations reported in Section 3 has been previously described [29]. We only reiterate a few important aspects here. It is an explicit chain, coarse-grained, structure based, non-additive model. To avoid excessive computational burden, our model is coarse-grained to the level of three atoms per residue and does not explicitly represent solvent molecules. Attractive interactions are dictated by the experimentally determined native structure and are of a uniform strength (independent of the amino acid identities). A consequence of the principle of minimal frustration [1] is that native contacts should be significantly more favorable than non-native contacts so that only those pairs of residues in contact in the experimentally determined native structure are assigned attractive interactions during the simulation. Although in reality non-native interactions are certainly present, their primary effect is to provide an additional source of friction, slowing the progression through the partially native manifold [30], [31]. Structure based models have generally shown agreement with a variety of protein folding experiments although there are a few systems such as Im7 where specific non-native effects are quite apparent [32]. In our model, non-additive forces are approximated by introducing a non-additivity exponent 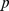 as shown in Equation 6, where 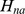 is the non-additive term in the Hamiltonian, 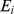 is a pairwise additive energy term and 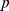 is the non-additivity exponent. For the current study, a value of 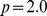 was used. Previous work indicates that adding a modest amount of non-additivity improves predictions of experimentally determined rate constants for both global and sub-global folding events of natural proteins [33], [34].
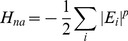
(6)


### 2.2 Example system

**Figure 1:**
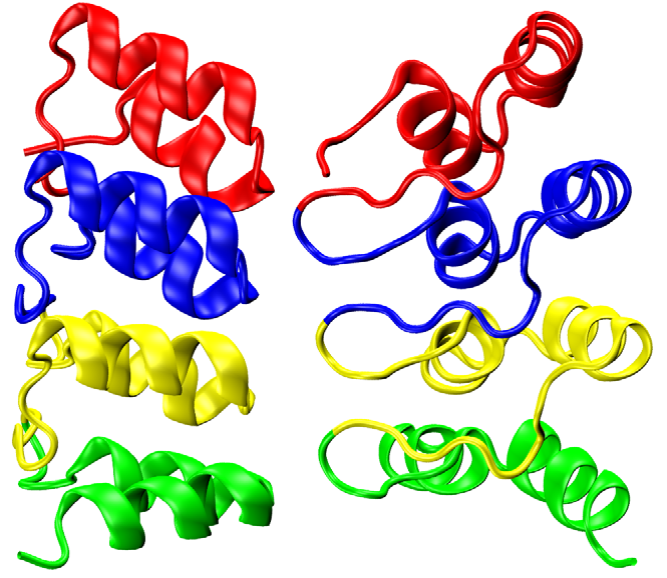
The protein 4ANK, comprised of 4 identical consensus ANK repeats. Each ANK is colored distinctly. The N-terminal repeat is colored red. The Visual Molecular Dynamics (VMD) software package [60] was used to visualize the structures in this work.

The ankyrin repeat (ANK) is a pervasive 33-residue motif found predominantly in eukaryotes [35]. It has been an excellent basis for constructing model systems for protein folding [16], [36]–[38] and engineering [39]–[44]. Through detailed comparison of ANK sequences, a consensus sequence – one that best represents the entire family – has been defined [15]. The secondary structure of a consensus ANK runs 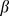-strand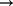 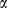-helix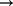 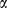-helix
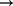
loop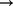 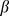-strand. The resulting tertiary structure contains a 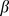-hairpin comprised of two rather short 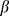 strands coming from the N- and C-terminal ends of consecutive repeats. Previous work has shown that single ankyrin repeats in isolation do not adopt stable tertiary structures [15]. Our example system, 4ANK (RCSB PDB [45] ID: 1N0R [15]), is shown in Figure 1. The short 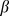-strands are shown as coil in this particular representation. Not all published coordinates of ANK proteins are annotated as having 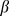-strands elements. However, these extended loops typically populate the 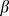-strand region of the Ramachandran plot. Variations in secondary structure detection algorithms (for example, consideration of hydrogen bonding geometry) may account for these apparent discrepancies.

Different groups have arrived at diverse descriptions of specific ANK protein folding mechanisms. Marchetti Bradley and Barrick, studying the Notch ankyrin domain (comprised of 7 ANK repeats), concluded that the central three ANKs of that protein formed the (early) transition state, based on 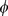 value analysis. [46] Ferreiro and coworkers, who computationally evaluated the folding of ANK proteins ranging from 3 to 7 repeats, concluded that the folding nucleus consists not of an integer number of repeats but of one ANK plus the first helix of the following ANK repeat [16]. In order to remain agnostic regarding the nature of the nucleus without introducing unnecessary complexity, we have chosen to characterize the foldon macrobasins at both the ANK and the half-ANK resolution. To avoid subtleties associated with how sequence differences between the repeats can change the folding mechanism, we have chosen to study a consensus ANK protein (containing identical repeats) and simulate a model with uniform stabilizing contact energies.

**Figure 2:**
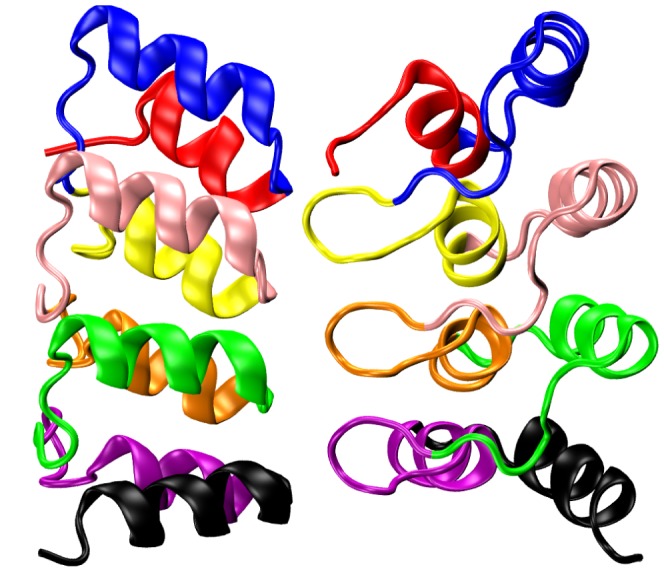
A structurally motivated foldon definition that splits each ANK element into two parts (8 foldons total). 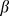
-hairpins are contained within a single foldon (odd-numbered foldons).

4ANK is a designed ANK protein consisting of three identical, consensus repeats followed by a fourth consensus ANK lacking its final 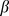-strand (which usually frays and promotes aggregation) [15]. A C-terminal tyrosine is the only non-consensus residue in the protein as constructed in the laboratory. Figures 1 and 2 show the experimentally determined structure of 4ANK and the two different foldon definitions we explored. One foldon definition assigns each ANK to its own foldon, while the second one divides the protein into 8 foldons of length 12, 19, 14, 19, 14, 19, 14, and 15 residues. The second definition was chosen so that the 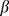-turn elements are contained within a single foldon. This allows us to monitor the formation of previously proposed [16] folding nuclei without deciding beforehand which ANKs would be involved.

## Results

**Figure 3:**
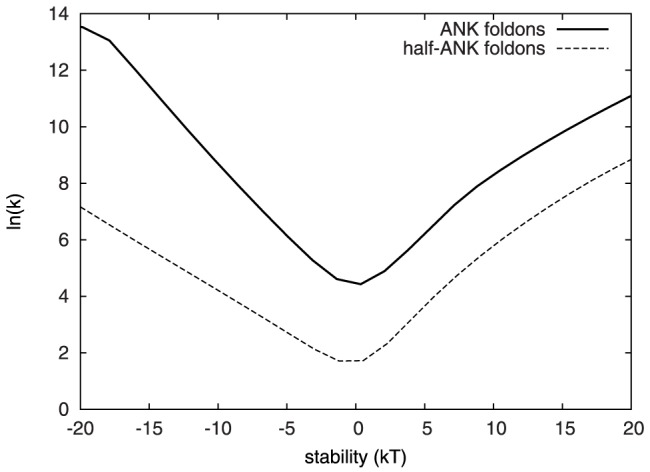
Thermal chevron plots obtained using two foldon definitions. For the ANK foldon definition (solid line) the maximum folding rate, at greatly stabilizing conditions, approaches the maximum downhill rate (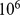 s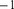). The minimum rate for the ANK definition is around 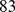 s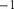, for the half-ANK definition, about 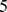 s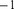.

In Figure 3 we show the calculated characteristic rate coefficients for the protein 4ANK as a function of the relative stability of the completely folded and completely unfolded macrobasins. At lower temperatures (more negative stabilities) the characteristic rate reflects the rate of formation of the folded state – this parallels the experimental scenario where denatured protein is rapidly equilibrated in stabilizing conditions. At higher temperatures (more positive stabilities), the unfolding process dominates the relaxation kinetics. The rates become smallest when the folding and unfolding rate eventually meet near the folding temperature. For strictly two-state folders (with a transition state that does not vary with the stability) this sort of 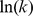 vs. stability plot has a sharp V-shape and is therefore called a “chevron plot”. Deviations from a strict V-shape are expected for folding mechanisms with intermediates. Experimentally, chevron plots are typically obtained by using chemical denaturant to change the relative stability of the folded and unfolded states. In computer models that lack an explicit representation of chemical denaturants, it is necessary to find other ways to change the relative stability of the folded and unfolded states, and temperature is a common choice. Although not guaranteed to behave identically, calculated thermal chevron plots have been fruitfully compared to experimental chemical denaturant chevron plots to shed light on specific questions related to real biological systems [47], [48].

Figure 3 shows chevron plots calculated using the ANK and half-ANK foldon definitions. Both foldon definitions give similar chevron plots although the rates obtained using the half-ANK definitions are lower. Using either foldon definition, the plots show curvature in the unfolding arm.

**Figure 4:**
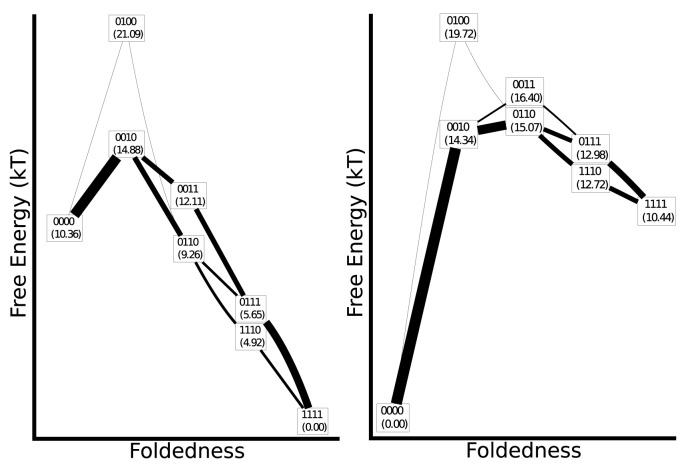
Flux diagrams for the full-ANK mechanism. The vertical coordinate approximates the free energy of each macrobasin and the precise relative free energies in units of 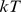 are given in parentheses. The horizontal coordinate approximates the global reaction coordinate. A line is drawn between each pair of connected macrobasins, and the width of the line is proportional to the flux. A minimum line width is enforced for clarity. Folding conditions are show on the left, unfolding on the right.

**Figure 5:**
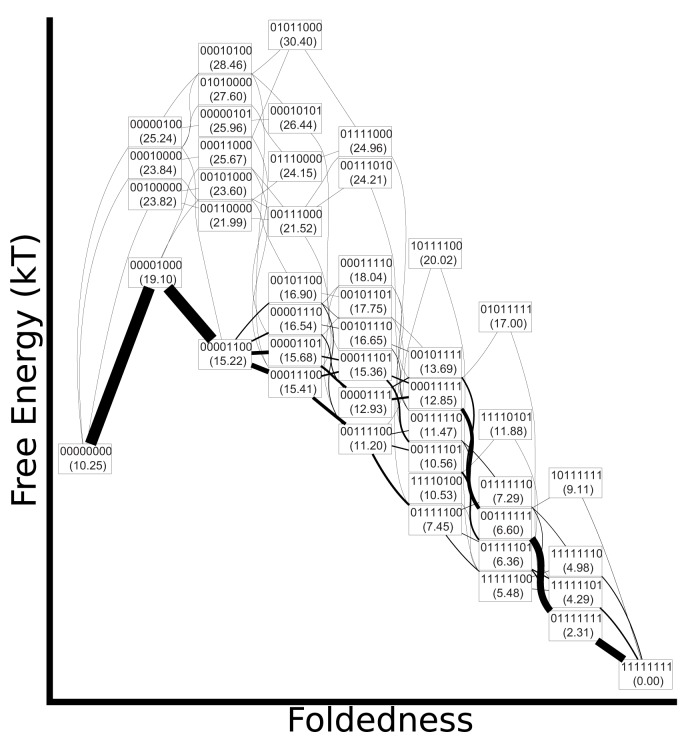
Folding flux diagram for the half-ANK mechanism. Symbols have the same meaning as in Figure 4.

**Figure 6:**
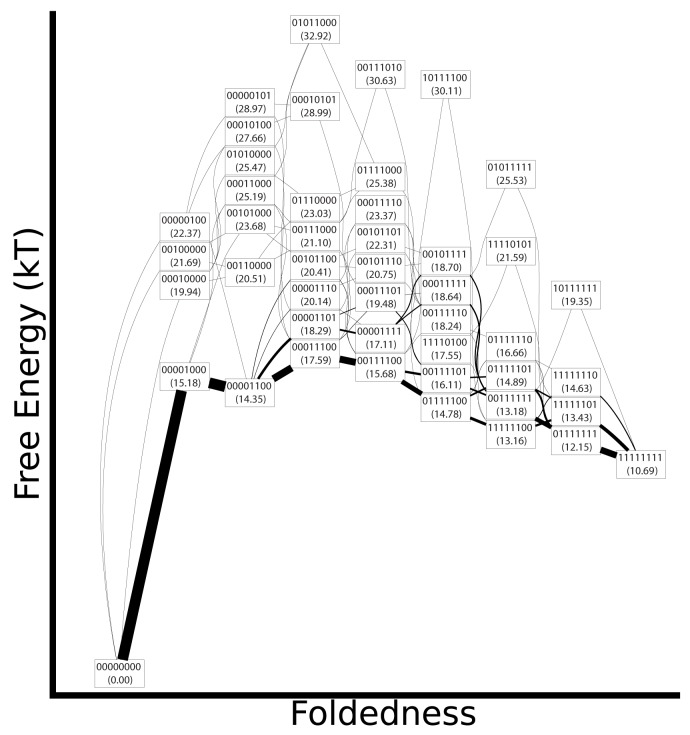
Unfolding flux diagram for the half-ANK mechanism. Symbols have the same meaning as in Figures 4 and 5.

For each foldon definition, we calculated the cumulative folding and unfolding fluxes using Equation 5 (Figures 4, 5 and 6). The relative stabilty of the folded and unfolded macrobasins was chosen to be in the range of 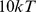 in all cases, about half way up the folding or unfolding arm. The flux calculation was started with 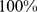 of the population in either the folded or unfolded state, and Equation 5 was evaluated at 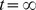, yielding the equilibrium fluxes.

The mechanism inferred using ANK foldons (Figure 4) goes through a transition state with the third repeat folded. At high folded state stability, folding continues downhill in free energy through several competing pathways. The unfolding mechanism at high temperature is approximately the reverse of the folding process at low temperature, but it differs in that a single pathway dominates, proceeding through a broad transition state that contains both the 0010 and 0110 macrobasins. In contrast to folding conditions, relatively little flux flows through 0011.

Figure 5 shows the fluxes for folding according to the half-ANK foldon definition. With 46 macrobasins sampled, the half-ANK mechanisms are more elaborate. Flux goes through multiple pathways that are closely related to each other and similar to the previously discussed pathways for the ANK foldon definition. Most of the flux goes through the macrobasin 00001000, which has the N-terminal helix of repeat 3 folded, and then through 00001100 to complete the folding of the 3rd repeat. While we predict a relatively high stability for the macrobasin 01111101, the flux analysis shows that this macrobasin is not kinetically significant. The mechanism does not follow trivially from the thermodynamics; the locality of transitions matters.

The unfolding fluxes under the half-ANK foldon assignment are shown in Figure 6. Unfolding is initiated at the termini. As with the ANK foldon case, the half-ANK mechanism goes through an intermediate with the center two repeats folded. For levels of global foldedness where an even number of half ANK units are folded, those macrobasins with all full ANK units either completely folded or unfolded (such as 00111100 and 00001111) are always found to be more stable than those with partially folded ANKs (such as 00011101 and 0010110). As a result, these states tend to have a larger fraction of the flux, although the exact amount of flux depends on the detailed connectivity of the model.

## Discussion

Kinetic equation formalisms are useful as a way of coarse-graining protein folding landscapes and extracting measurable kinetics [10], [11], [49], [50]. Here we develop an approach wherein umbrella sampling over a global folding reaction coordinate allows for accurate quantification of the free energies of the folding intermediates. A similar method was used by Ferreiro *et al.* to study TPR repeat proteins [17]. The current method extends that work by calculating folding kinetics and fluxes using simple assumptions about the kinetic connectivity of the network of intermediates and the universal rate for downhill transitions between macrobasins.

Curvature in the unfolding arm of chevron plots is a well studied phenomenon [51]–[53]. Experimental studies also have shown that ANK proteins have substantial curvature in the unfolding arm of the chevron plot [38], [46], [54]–[57] in qualitative agreement with the present model's prediction. Although some simple coarse-grained models show large amounts of rollover in the folding and unfolding arms of calculated chevron plots, previous theoretical work [58], [59] has shown that these effects are lessened when physically plausible many-body interactions are included, as they are in the current study. The inferred mechanisms are consistent with the notion that consensus ANK proteins, which lack energetic biases that result from sequence heterogeneity between repeats, are likely to fold through an “inside-out” mechanism, with the central repeats nucleating folding. While specific folding pathways occur, which ones dominate clearly depends on the conditions under which the folding or unfolding occurs. Also, the resolution at which kinetics is monitored may determine whether a single pathway may appear to be dominant or whether multiple pathways can be discerned.
