# Correction: Discrete Kinetic Models from Funneled Energy Landscape Simulations

**DOI:** 10.1371/annotation/16c11a12-4245-403c-80ab-e6662baf16cd

**Published:** 2013-05-24

**Authors:** Nicholas P. Schafer, Ryan M. B. Hoffman, Anat Burger, Patricio O. Craig, Elizabeth A. Komives, Peter G. Wolynes

There were errors in the author affiliations. The correct versions of the affiliations are available below:

Nicholas P. Schafer^2,5☯^, Ryan M. B. Hoffman^1,2☯^, Anat Burger^2,3^, Patricio O. Craig^1,2^, Elizabeth A. Komives^1^, Peter G. Wolynes^2,4*^

1 Chemistry/Biochemistry, University of California San Diego, La Jolla, California, United States of America,

2 Center for Theoretical Biological Physics, University of California, San Diego and Rice University, La Jolla, CA and Houston, TX, USA

3 Physics, University of California San Diego, La Jolla, California, United States of America,

4 Chemistry, Rice University, Houston, Texas, United States of America,

5 Physics and Astronomy, Rice University, Houston, Texas, United States of America

In numerous occasions in the References section, the author name "Wolnes P" should have been "Wolynes PG". 

